# Secondary Hyperparathyroidism and Hypertension: An Intriguing Couple

**DOI:** 10.3390/jcm9030629

**Published:** 2020-02-27

**Authors:** Mariadelina Simeoni, Alessandra F. Perna, Giorgio Fuiano

**Affiliations:** 1 Department of Translational Medical Sciences, University of Campania ‘Luigi Vanvitelli’, 80131 Naples, Italy; alessandra.perna@unicampania.it; 2 Department of Medical and Surgery Sciences, ‘Magna Graecia University’, 88100 Catanzaro, Italy; fuiano@unicz.it

**Keywords:** secondary hyperparathyroidism, hypertension, hemodialysis, calcimimetic

## Abstract

Secondary hyperparathyroidism (SHPTH) is a major complication in patients on maintenance hemodialysis burdened with high cardiovascular risk. Hypertension is also a high prevalence complication contributing to an increase in the mortality rate in hemodialysis patients. A possible association between SHPTH and hypertension has been widely reported in the literature and several pathogenetic mechanisms have been described. There is evidence that the decrease of plasma iPTH levels are correlated with hypertension correction in hemodialysis patients undergoing parathyroidectomy and oral calcimimetics administration. We have observed a similar behaviour also in a patient on chronic hemodialysis treated with Etelcalcetide. Even if this is an isolated observation, it could stimulate future investigation, possibly in dedicated clinical trials.

## 1. Introduction

**Figure 1:**
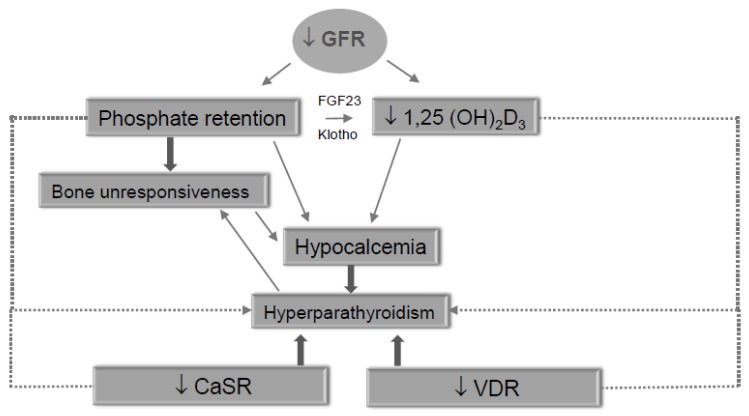
Pathogenesis flow-chart of SHPTH development in CKD.

Secondary hyperparathyroidism (SHPTH) is a universal complication of chronic kidney disease (CKD) affecting more than 80% of hemodialysis patients [1,2]. This complication is particularly relevant since CKD is already burdened by a significant cardiovascular risk increase and quality of life worsening [3]. Pathophysiologic mechanisms leading to SHPTH start in early CKD stages and increase in direct correlation with renal function loss [4,5,6]. Decreased active Vitamin D production, hypocalcemia and hyperphosphatemia are common alterations in CKD and represent a continued trigger on parathyroid glands to increase iPTH production. Additional factors contribute as well [7]. The persistence of these triggers leads to a progressive reduction of calcium-sensing receptors (CaSR) and vitamin D receptors (VDR) expression in parathyroid glands preluding to irreversible parathyroid gland hyperplasia onset [8] (Figure 1). This histological change is particularly relevant since patients on hemodialysis with tertiary hyperthyroidism are more likely to develop cardiovascular events and bone fractures with quality of life worsening and increased healthcare costs and mortality [2]. In SHPTH, in fact, normal calcium deposition in bone tissue is diverted into vascular wall inducing atherosclerosis worsening [9].

Hypertension is another relevant and highly prevalent cardiovascular risk factor in hemodialysis patients, as widely reported in the literature [10,11,12,13,14]. Different hypertensive mechanisms have been described in this patient category. Indeed, fluid overload, dialysate calcium content and renin-angiotensin hyperactivity are the most frequent causes of high blood pressure (BP) in patients on extracorporeal renal replacement therapy [15]. However, other hypertensive mechanisms have also been described. A possible pathophysiological cross-link between hypertension and hyperparathyroidism has been widely reported with evidence of BP normalization after parathyroidectomy [16,17]. Thus, hyperparathyroidism-related hypertensive mechanisms in hemodialysis patients have been investigated with interesting results. In fact, both the role of alterations of calcium distribution and direct hypertensive activities induced by PTH have been discovered and described [18].

Despite the association of SHPTH and hypertension being very frequent in hemodialysis patients with a possible exponential increase of cardiovascular risk [19], these two clinical entities are still approached in a separate manner by nephrologists. On the contrary, a revaluation of this behavior should be addressed.

This literature analysis is aimed to provide an updated overview of what is known on the pathophysiological basis of the SHPTH and hypertension association. Moreover, a discussion on novel therapeutic approaches to SHPTH and their impact on blood pressure will include our experience on a single clinical case of blood pressure normalization in a hemodialysis patient with SHPTH treated with Etelcalcetide.

## 2. Main Text

### 2.1. The Pathogenetic Cross-Link between Hypertension and Hyperparathyroidism

**Figure 2:**
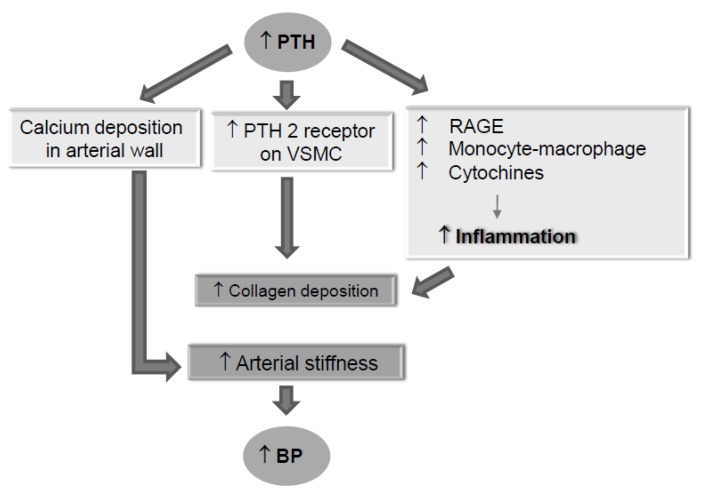
Mechanisms involved in hyperparathyroidism-related blood pressure increase. *VSMC: vascular smooth muscle cell*.

The suspicion for the existence of a pathophysiological relationship between hyperparathyroidism and blood pressure increase has far-away origins. In 1996, Goldsmith et al. published the results of a study conducted on 21 hypertensive hemodialysis patients with tertiary hyperparathyroidism that had undergone parathyroidectomy (PTX) [16]. Interestingly, after the surgical procedure, BP values were significantly reduced together with mean plasma calcium levels and mean heart rate. However, post-surgery iPTH decrease was not followed by a prompt BP normalization, that was instead observed a few months later. Consequently, Goldsmith et al. hypothesized that this effect was linked to a long-term corporeal re-arrangement of calcium distribution. This hypothesis has been sustained and confirmed by other authors describing a mechanism by which, following the post-surgery iPTH lowering, the removal of calcium from the vessel walls might explain a blood pressure improvement related to vessel stiffness reduction [20]. Several other recent studies investigating this important issue have also reported direct iPTH effects involved in vascular stiffness. Specifically, iPTH stimulates PTH2 receptors expressed on vascular smooth muscle cells with a consequent increase of collagen production [19]. Other iPTH related pro-atherosclerosis mechanisms have been also described. They involve an increase in both receptors of advanced glycation end products (RAGE) expression and monocyte-macrophages cytokines and IL-6 production [21] (Figure 2). Interestingly, other reports have evidenced that iPTH is also able to influence both contractile and chronotropic activity of myocardial cells. Specifically, high blood iPTH levels induce ventricular hypertrophy accompanied by a reduction of myocardial contractility and an increase in heart rate [22,23]. A hypertensive role of up-regulated FGF23 in response to hyperphosphatemia in SHPTH patients has also been supposed. In fact, besides the known contribution of FGF23 to calcium deposition in the vascular wall [24], an important mechanism leading to FGF23-related blood pressure increase has been described by Andrukhova et al. They reported that the up-regulating influence of FGF23 on Na^+^-Cl^−^ transporter expression in convoluted distal tubule is at the basis of a sodium-retentive hypertensive mechanism [25].

As for several reports, structural and hemodynamic pathologic changes associated to SHPTH appear to be partially reversible, since an improvement of arterial stiffness and survival has been observed after PTX in patients that also achieved normal serum calcium levels [26,27]. Numerous other reports have been published since, and recently a metanalysis of eight studies including 94 hemodialysis patients [28,29,30,31,32,33,34] on this focus was published by Giorgina Piccoli’s group. Although based on a limited number of patients, the metanalysis study results evidenced a reduction of both systolic and diastolic BP in response to PTX [17]. The significance of this beneficial effect was more evident on systolic blood pressure leading to a confirmation of the hypothesis that BP changes after hyperparathyroidism correction are linked to vascular stiffness reduction. Indeed, further investigations on pathophysiologic mechanisms underlying the cross-link between hyperparathyroidism and hypertension in patients on renal replacement therapy are strongly desirable. The full confirmation of a beneficial effect of hyperparathyroidism correction in larger populations is also needed.

### 2.2. Past and Future Therapeutic Implications

As described in the previous paragraph, data on the beneficial effects of PTX on BP control in hemodialysis patients are limited, but undoubtedly current evidence highlight an association between hyperparathyroidism and hypertension. Little is known, however, about the effect of secondary hyperparathyroidism therapy on BP.

Besides the indisputable role of phosphate chelating agents, vitamin analogues in the control of iPTH secretion, the advent of calcimimetic agents has profoundly changed and improved the therapeutic approach to SHPTH.

Cinacalcet has been the first available calcimimetic agent [35]. The EVOLVE study is the largest clinical trial testing this oral drug vs. placebo. In the EVOLVE study, 3883 hemodialysis patients with SHPTH were enrolled to receive a 21-month treatment course with Cinacalcet. Only the Cinacalcet group showed a significant iPTH blood levels reduction [36], however, in the absence of any beneficial modification of mortality and hospitalization rates [37]. Conversely, both systolic and diastolic BP showed a significant reduction only in the group treated with the calcimimetic [37]. In line with calcium-based pathophysiological hypothesis, it can be supposed that the tendency to hypocalcemia associated with Cinacalcet administration might represent the mechanism by which BP reduction occurred. On the other hand, Cinacalcet is often accompanied by a corollary of gastrointestinal symptoms.

Etelcalcetide is a novel intravenous calcimimetic agent approved by the FDA in 2017 for the treatment of SHPTH in adult patients with chronic kidney disease (CKD) on hemodialysis.

This approval was largely based on positive results from two randomized, double-blind, placebo-controlled Phase 3 studies [38]. An aggregate of 1023 patients enrolled in the two trials was randomized to receive intravenous Etelcalcetide or placebo three times a week, at the end of their dialysis sessions in addition to a standard of care that could include vitamin D analogues and/or phosphate binders. The primary endpoint of both studies was the proportion of patients achieving greater than 30% PTH reduction from baseline within the first 20 treatment weeks corresponding to the Efficacy Assessment Phase (EAP). Secondary endpoints included both the proportion of patients achieving PTH ≤ 300 pg/mL during the EAP and the reduction of albumin-adjusted calcium (cCa), phosphate (P), PTH and cCa x P achieved during the EAP.

In both studies, the primary end-point was achieved in a significantly higher percentage of patients in the Etelcalcetide group compared to controls. Similarly, a greater proportion of patients within the treatment group compared to the placebo, reached the secondary end-points during the EAP.

In a pooled analysis of the two Phase 3 placebo-controlled studies, as expected, asymptomatic and symptomatic hypocalcemia occurred more frequently in patients treated with Etelcalcetide compared to placebo. Other commonly reported adverse reactions were muscle spasms, diarrhea, nausea, vomiting, headache, and paresthesia/hypoesthesia. The impact of Etelcalcetide on blood pressure was instead not explored.

In the first trial exploring the impact of Etelcalcetide vs. Cinacalcet on iPTH, Etelcalcetide resulted more efficacious in lowering iPTH, but also, in this case, no significant differences were reported relating to blood pressure. However, mean baseline BP in both studied groups was normal and indeed, this did not stimulate a secondary analysis. However, it is noticeable that in the Etelcalcetide group, a higher percentage of patients (6.8%) than in the Cinacalcet group (2.9%) showed hypotension within common side adverse effects. Even the number of hypocalcemia episodes was higher in the Etelcalcetide group [39]. As detailed in Table 1, a similar tendency has been observed in all trials assessing the efficacy and safety of Etelcalcetide. However, in the absence of a secondary analysis, even in these studies, a noteworthy effect of Etelcalcetide on hypertension was not found [40,41].

### 2.3. Our Experience

**Figure 3:**
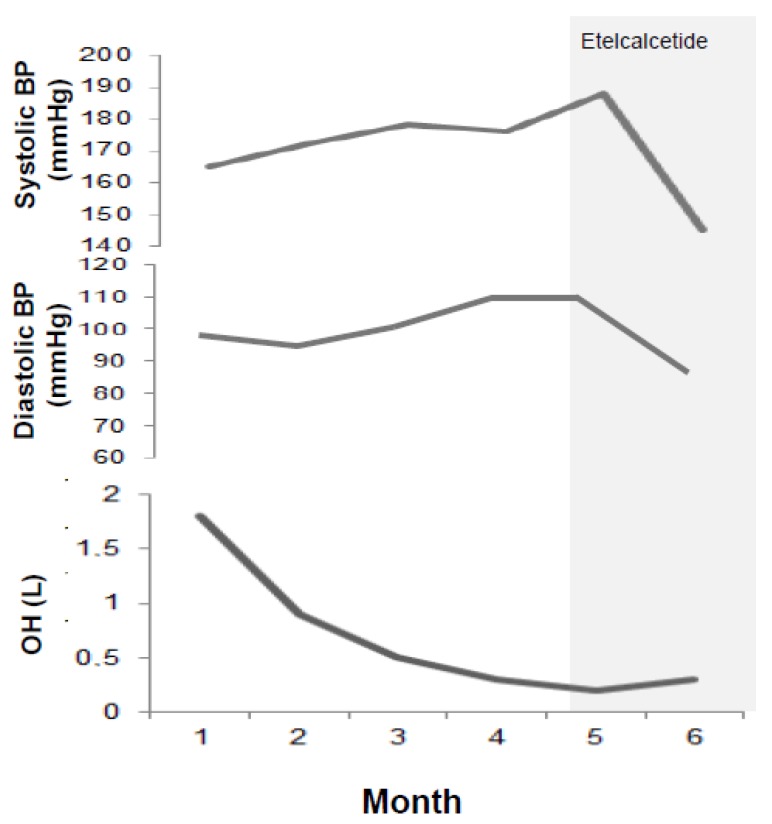
Unrelated blood pressure response to dry weight reduction in our patient before starting Etelcalcetide. *OH: overhydration at body composition monitoring (BCM)*.

**Figure 4:**
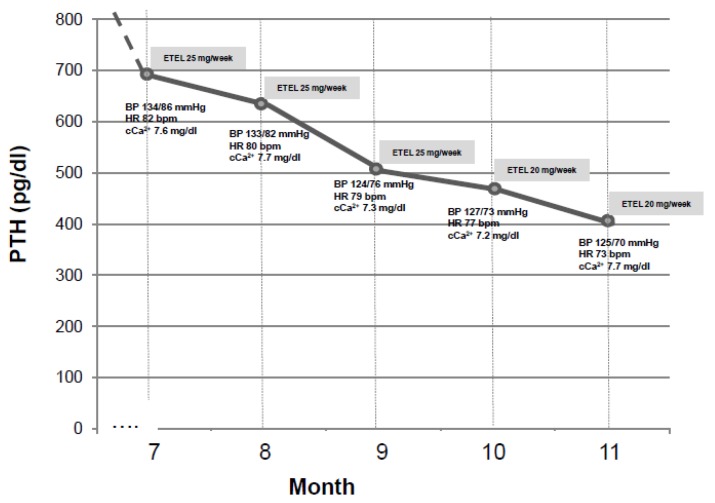
Variations of blood pressure, heart rate, albumin corrected-serum calcium in response to iPTH reduction induced by ETEL (Etelcalcetide).

We recently experienced the effect of Etelcalcetide on blood pressure in a 37 years old young male patient on chronic hemodialysis in the past three years. This patient was complicated with SHPTH in treatment with Cinacalcet 30 mg/die (orally administered by the nurse at the end of each dialysis session) and had baseline iPTH 421 pg/mL. At the time, his home therapy also included Lanthanum carbonate 1 g twice/day, Irbesartan 150 mg/day and Omeprazole 20 mg/day. So far, blood pressure was well controlled and stable during hemodialysis sessions. In January 2018, a sudden increase in intradialytic blood pressure and heart rate (mean systolic blood pressure was 190/110 mmHg and mean heart rate was 110 bpm) was accompanied by a significant increase in iPTH, (peak value was 1200 pg/mL). An electrocardiogram and an echocardiogram were performed at that time, not showing any noteworthy change except for a sinusal tachicardia and previously known ventricular hypertrophy. The uncontrolled hypertension was resistant to both pharmacological therapy increase (Amlodipine 10 mg/day, Clonidine TTS 2.5 mg/week were consecutively added) and dry weight reduction according to bioimpedentiometry results (Figure 3). Anti-RAAS agents were not added because of the tendency to hyperkalemia [42,43]. After tertiary hyperparathyroidism was excluded by a Technetium 99m-MIBI-SPECT scan, Cinacalcet was discontinued and Etelcalcetide was started at the standard dose of 5 mg to be administered intravenously at the end of each dialysis session. This dose was thereafter increased to a maximum of 7.5 mg three times per week. In the following six months, we assisted to a progressive and synchronous blood pressure and heart rate normalization and a significant iPTH reduction to baseline values (see Figure 4). This is an anecdotic case increasing the evidence for a cross-link between hypertension and SHPTH and indicating a possible synergistic hypotensive effect by Etelcalcetide in our patient. It would be important to clarify if this effect is ubiquitous in patients treated with Etelcalcetide and the underlying mechanisms.

## 3. Conclusions

A possible association between SHPTH and hypertension in hemodialysis patients has been widely reported in the literature and several pathogenetic mechanisms have also been disclosed [16,17,18,19,20]. A hypertension correction has been described following the decrease of iPTH plasma levels in hemodialysis patients undergone to parathyroidectomy [16,17,28] and oral calcimimetics administration [36]. We have observed a blood pressure normalization in a patient on chronic hemodialysis in which SHPTH was treated with Etelcalcetide. This is a single observation, however, it might stimulate future investigations possibly in dedicated clinical trials.

## Figures and Tables

**Table 1:** Studies testing Etelcalcetide in comparison to placebo or Cinacalcet for SHPTH correction in hemodialysis patients in which hypotension resulted to be a common side effect. *ETEL = Etelcalcetide; SAE = Side adverse event*.

Study ID	Study Design	*n*	Intervention vs. Comparator	Follow-Up (Weeks)	Outcome −30% iPTH Reduction (% pts)		SAE Hypocalcemia *n*		SAE Hypotension *n*	
					ETEL	COMPARATOR	ETEL	COMPARATOR	ETEL	COMPARATOR
Block G.A. et al. JAMA 2017	2 Phase III RCT (A-B)	1023	Etelcalcetide vs. Placebo	26	74.6	8.9	35	1	31	29
Block G.A. et al.; PLOS One 2019	Pooled data from 5 RCT	3005	Etelcalcetide vs. Cinacalcet or Placebo	Mixed (26-52-extended)	71.4	33.3	52	9	53	36
Block G.A. et al.; JAMA 2017	RCT	683	Etelcalcetide vs. Cinacalcet	26	68.2	57.7	17	8	23	10
Bushinsky D.A. et al.; NDT 2019	RCT	158	‘Switch’ study from Cinacalcet to Etelcalcetide	52	68		33	35	75	30
